# Supplementary material for: Development of a Dental Implantable Temperature Sensor for Real-Time Diagnosis of Infectious Disease
Source: Sensors (Basel). 2020 Jul 16;20(14):3953. doi: 10.3390/s20143953 (PMC7412512; doi:10.3390/s20143953)
Supplement: Supplementary file 1 [file sensors-20-03953-s001.zip › sensors-847948-supplementary/Figure S1 with description.pdf]

Figure S1. Biocompatibility of polyimide (PI)

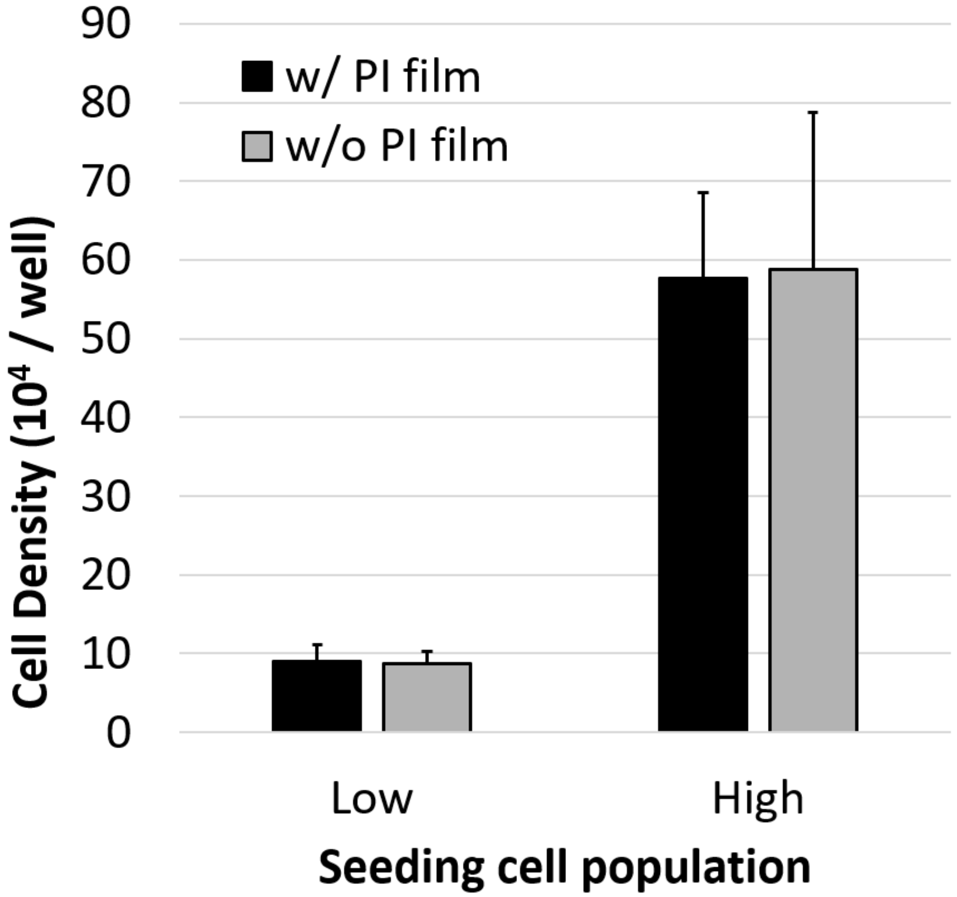

To test the biocompatibility of a photo-definable polyimide (HD-8820) film, we cultured mouse epithelial tongue cells with or without the film in 6-well culture dishes up to 5 days. The cells exhibited good affinity and normal morphology on the polyimide film, as well as on the culture dish. In addition, no toxic effects such as abnormal proliferation and uneven population were monitored based on daily microscopic observations from 19 samples. After 5 day culture, viable cells which adhered well on the surface were counted and we plotted the density in Figure S1. In the case of a high seeding population, the variance of cell density was greater than in the case of a low seeding population. However, there was no statistical difference between the cell density with and without the polyimide film in the culture. Therefore, the polyimide film is biocompatible and nontoxic
